# The mannose receptor LY75 (DEC205/CD205) modulates cellular phenotype and metastatic potential of ovarian cancer cells

**DOI:** 10.18632/oncotarget.7288

**Published:** 2016-02-09

**Authors:** Adnen Faddaoui, Magdalena Bachvarova, Marie Plante, Jean Gregoire, Marie-Claude Renaud, Alexandra Sebastianelli, Stephane Gobeil, Chantale Morin, Elizabeth Macdonald, Barbara Vanderhyden, Dimcho Bachvarov

**Affiliations:** ^1^ Department of Molecular Medicine, Université Laval, Québec, Canada; ^2^ Centre de recherche du CHU de Québec, L'Hôtel-Dieu de Québec, Québec, Canada; ^3^ Department of Obstetrics and Gynecology, Université Laval, Québec, Canada; ^4^ Centre de recherche du CHU de Québec, CHUL, Québec, Canada; ^5^ Department of Cellular and Molecular Medicine, University of Ottawa, Ottawa, Canada

**Keywords:** LY75 (DEC205), epithelial ovarian cancer, epithelial-to-mesenchymal transition, intraperitoneal xenografts, shRNA & CRISPR/Cas9

## Abstract

The molecular basis of epithelial ovarian cancer (EOC) dissemination is still poorly understood. Previously, we identified the mannose receptor LY75 gene as hypomethylated in high-grade (HG) serous EOC tumors, compared to normal ovarian tissues. LY75 represents endocytic receptor expressed on dendritic cells and so far, has been primarily studied for its role in antigen processing and presentation. Here we demonstrate that LY75 is overexpressed in advanced EOC and that LY75 suppression induces mesenchymal-to-epithelial transition (MET) in EOC cell lines with mesenchymal morphology (SKOV3 and TOV112), accompanied by reduction of their migratory and invasive capacity *in vitro* and enhanced tumor cell colonization and metastatic growth *in vivo*. LY75 knockdown in SKOV3 cells also resulted in predominant upregulation of functional pathways implicated in cell proliferation and metabolism, while pathways associated with cell signaling and adhesion, complement activation and immune response were mostly suppressed. Moreover, LY75 suppression had an opposite effect on EOC cell lines with epithelial phenotype (A2780s and OV2008), by directing epithelial-to-mesenchymal transition (EMT) associated with reduced capacity for *in vivo* EOC cell colonization, as similar/identical signaling pathways were reversely regulated, when compared to mesenchymal LY75 knockdown EOC cells.

To our knowledge, this is the first report of a gene displaying such pleiotropic effects in sustaining the cellular phenotype of EOC cells and points to novel functions of this receptor in modulating EOC dissemination. Our data also support previous findings regarding the superior capacity of epithelial cancer cells in metastatic colonization of distant sites, compared to cancer cells with mesenchymal-like morphology.

## INTRODUCTION

Epithelial ovarian cancer (EOC) accounts for 4% of all cancers in women and is the leading cause of death from gynecologic malignancies [1]. Despite treatment improvements, long-term survival rates for patients with advanced disease remain disappointing [2]. The molecular basis of EOC initiation and progression is still poorly understood. Previously, we have applied an epigenomics approach to investigate the possible implication of aberrant DNA methylation in EOC etiology [3]. This approach led to the identification of novel EOC oncogenes, potentially modulated by epigenetic mechanisms (hypomethylation) in advanced EOC [4–7]. The antigen receptor LY75 (also known as DEC205/CD205 and gp200-MR6) was also among the genes identified to be notably hypomethylated in high-grade (HG) serous EOC tumors.

LY75 is a type I transmembrane surface protein that belongs to the macrophage mannose receptor (MMR) family of C-type lectin endocytic uptake receptors, which also includes the mannose receptor, the M-type phospholipase A2 receptor (PLA2R) and Endo180/uPARAP [8]. LY75 is predominantly expressed by the thymic cortical epithelium and by dendritic cells (DCs) [9–12], but later was also detected on T and B lymphocytes and several other tissues/cell types, including intestinal epithelia and the epithelia of pulmonary airways, the stroma of the bone marrow and the capillaries of the brain [13]. Similar to the other MMR members, LY75 represents a single polypeptide chain receptor with a short cytoplasmic domain containing functional endocytosis motifs that direct its internalization through clathrin-coated pits to endosomes for major histocompatibility complex (MHC) loading and subsequent immune responses. The LY75 extracellular domain consists of an N-terminal cysteine-rich domain, a fibronectin type II domain, and ten C-type lectin domains that lack carbohydrate-binding function, and its natural ligands have not been still identified [8]. Yet, it is clear that LY75 plays an important role in antigen uptake for presentation and cross-presentation to T cells and the initiation of the immune response [14]. Accordingly, loading of LY75 with antigens is widely used in vaccination including cancer immunotherapy [15]. This approach has been exploited *in vitro* and *in vivo*, both in mice and humans, to deliver specific antigens to DCs, and induces specific CD81 and CD41 T-cell responses [16–23]. However, while the potential of LY75 as immunomodulatory target is clear, its biological role and the functional significance of its broad expression require a greater understanding.

In this study, we identified LY75 as a major modulator of the EOC cells phenotype. Suppression of LY75 induces mesenchymal-to-epithelial transition (MET) in EOC cell lines with mesenchymal morphology, accompanied by reduction of their migratory and invasive capacity *in vitro* and enhanced tumor cell colonization and metastatic growth *in vivo* in intraperitoneal (IP) xenograft EOC model. Surprisingly, LY75 knockout also leads to epithelial-to-mesenchymal transition (EMT) of EOC cells with epithelial phenotype, associated with decrease of their metastatic potential *in vivo*. Our findings are indicative for novel functions of this mannose receptor associated with EOC etiology and support the enhanced capacity of epithelial cancer cells in metastatic colonization of distant sites.

## RESULTS

### Analysis of LY75 expression in EOC tumors

**Figure 1:**
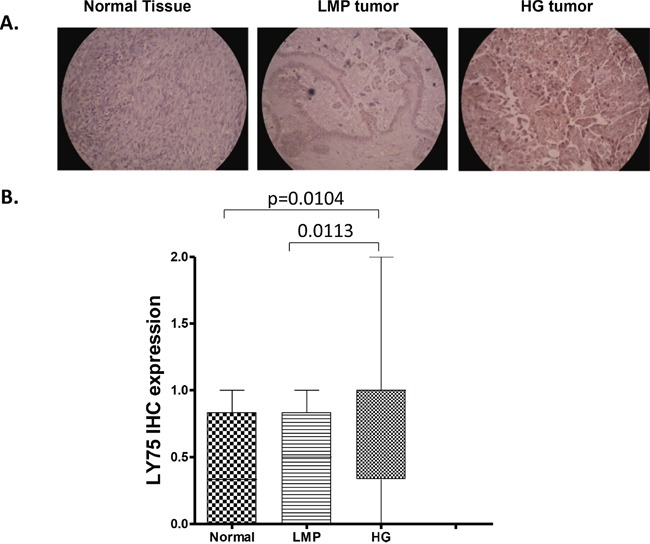
Analysis of LY75 expression in serous EOC tumors by IHC **A.** Representative IHC images of LY75 protein expression in normal ovarian tissues, low-malignant potential (LMP) tumors and high-grade (HG) tumors. **B.** Box-plot presentation of LY75 protein expression levels in normal ovarian tissues, LMP tumors and HG tumors.

We initially evaluated LY75 protein expression by immunohistochemistry (IHC) in numerous serous EOC tumor and normal ovarian tissue samples, using tissue microarrays (TMAs). Our TMAs included triplicate cores of 117 serous EOC tumors, including 13 low-malignant potential (LMP) tumors and 104 HG ovarian tumors. Thirteen normal ovarian tissue samples were also included as controls. Supplemental Table 1 shows the major clinical characteristics of these patients for whom extensive follow-up clinical data (up to 5-years) were available. The age ranged from 41 to 83 years (median: 66 years). High-grade tumors were all grade 3 (100%) including stage III (69%) and stage IV (31%) tumors. The majority of patients (93%) received a combination of platinum and paclitaxel. The median baseline CA125 was around 800. Forty percent of the patients had a progression or a recurrence within the first 6 months of follow-up; for 39 % of the patients the progression-free survival (PFS) interval was in the range of 7 to 24 months, and 21 % of the patients displayed PFS values higher than 25 months. As seen in Figure 1, LY75 displayed significantly higher expression only in HG serous EOC tumors compared to normal tissues (p = 0.0104), which possibly correlates with the LY75 hypomethylation status in advanced disease. Indeed, alternative methylation analysis (using the bisulfite sequencing PCR, or BSP approach) in independent set of serous HG serous EOC tumors confirmed the hypomethylation status of the putative LY75 promoter region in the tumor samples compared to normal ovarian tissues (Supplemental Figure 1), which is indicative for possible implications of epigenetic mechanisms in the control of LY75 expression in EOC. However, we did not observed any significant differences between the levels of LY75 expression and patients' PFS values (p = 0.622; see Supplemental Figure 2), which suggests that staining intensity for LY75 in pre-treatment surgical EOC specimens is not predictive of PFS.

### LY75 suppression directs MET in EOC cell lines with mesenchymal-like morphology

Next, we decided to verify if shRNA-mediated LY75 gene knockdown could produce any cancer-related phenotypic changes in EOC cells. Two shRNA constructs (#57363 and #57364), targeting different regions of the LY75 mRNA, were used for stable LY75 knockdown in the EOC cell line SKOV3, as this cell line was among the EOC cell lines displaying strong endogenous LY75 expression (see Supplemental Figure 3A). Clone selection for further analyses was based on qPCR and Western blot validation of the LY75 mRNA/protein expression levels in selected clones, compared to empty vector-transfected clones. Among the clones analyzed, the shRNA-LY75 knockdown clone 3 (sh-S3, obtained with shRNA construct #57363) and the shRNA-LY75 knockdown clone 6 (sh-S6, obtained with shRNA construct #57364), displayed significant decrease of LY75 mRNA and protein expression levels compared to the mock-transfected control (Supplemental Figure 3B and 3C), and were selected for further analyses.

**Figure 2:**
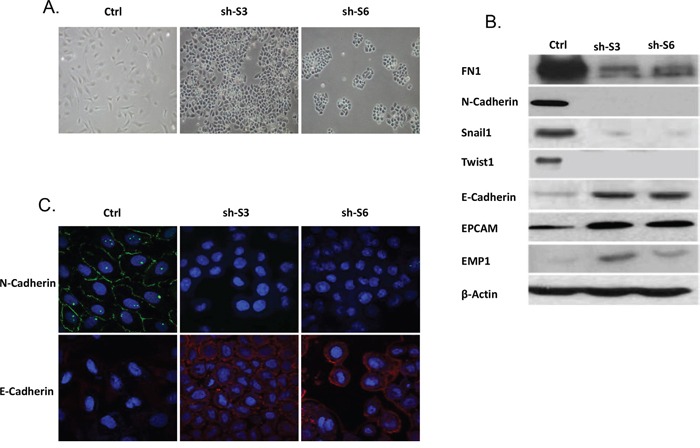
LY75 suppression directs MET in SKOV3 cells **A.** Representative phase-contrast images of SKOV3 control and LY75 knockdown clones (sh-S3 and sh-S6). **B.** Western blot analysis of the expression of different EMT (epithelial and mesenchymal) markers in the control and the LY75 knockdown SKOV3 clones. **C.** Immunofluorescence staining of E-cadherin and N-cadherin in the control and the LY75 knockdown SKOV3 clones.

SKOV3 represents a serous EOC cell line displaying mesenchymal (spindle-like) phenotype, including no evidence of cell-cell adhesion [24]. Remarkably, LY75 suppression induced MET in this cell line, as both sh-S3 and sh-S6 SKOV3 clones exhibited a typical epithelial phenotype with cuboid shaped cells forming discrete clusters, indicative for tight junctions (Figure 2A). This was further confirmed by analyzing the expression of specific EMT markers in both control and LY75 knockdown clones. Indeed, we observed a significant increase of the expression of E-cadherin, EPCAM and EMP1 (epithelial markers) in the sh-S3 and sh-S6 SKOV3 clones compared to the control, while the expression of the mesenchymal markers N-cadherin, TWIST1, FN1 and SNAIL1 were strongly down-regulated (Figure 2B). The same results were later obtained following CRISPR/Cas9-mediated LY75 knockout in SKOV3 cells (Supplemental Figure 4). Immunofluorescence analysis of the E- and N-cadherin expression in the control and LY75 knockdown clones supported the above findings, as N-cadherin was exclusively expressed in the control clone, while E-cadherin displayed expression only in the LY75 knockdown clones (Figure 2C).

**Figure 3:**
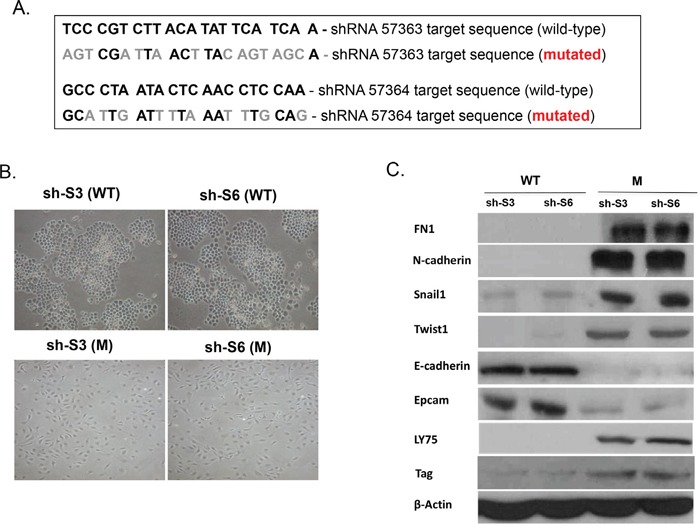
Ectopic expression of the wild-type (WT) and the mutated (M) LY75 gene in the LY75 knockdown SKOV3 clones sh-S3 and sh-S6 **A.** The LY75 cDNA mutations are shown, which modify the target sequences for the two shRNAs used (#57363 and #57364), without altering the LY75 protein sequence. **B.** Representative phase-contrast images of LY75 knockdown clones sh-S3 and sh-S6 upon ectopic expression of the wild-type (WT) or the mutated (M) LY75 gene. **C.** Western blot analysis of the expression of LY75 and different EMT (epithelial and mesenchymal) markers in the LY75 knockdown clones sh-S3 and sh-S6 upon ectopic expression of the WT or the M LY75 gene.

To further confirm the role of LY75 in determining the mesenchymal phenotype of SKOV3 cells, we re-expressed a mutated variant of the LY75 gene (cloned in the pcDNA3.1-DYK expression vector) in both our LY75 knockdown clones (sh-S3 and sh-S6). In this construct, the LY75 cDNA was mutated at the target sequences for the two shRNAs used (#57363 and #57364), without altering the LY75 protein sequence (see Figure 3A). The pcDNA3.1-DYK vector expressing the wild type (WT) LY75 cDNA was used as a control. As shown on Figure 3B, only the mutated LY75 construct induced LY75 protein expression in the LY75 knockdown clones. More importantly, LY75 re-expression completely restored the mesenchymal morphology in both LY75 knockdown clones sh-S3 and sh-S6 (Figure 3B), which was accompanied with re-establishing the parental SKOV3 expression pattern of the mesenchymal markers N-cadherin, TWIST1, FN1 and SNAIL1 and the epithelial markers E-cadherin and EPCAM (Figure 3C).

**Figure 4:**
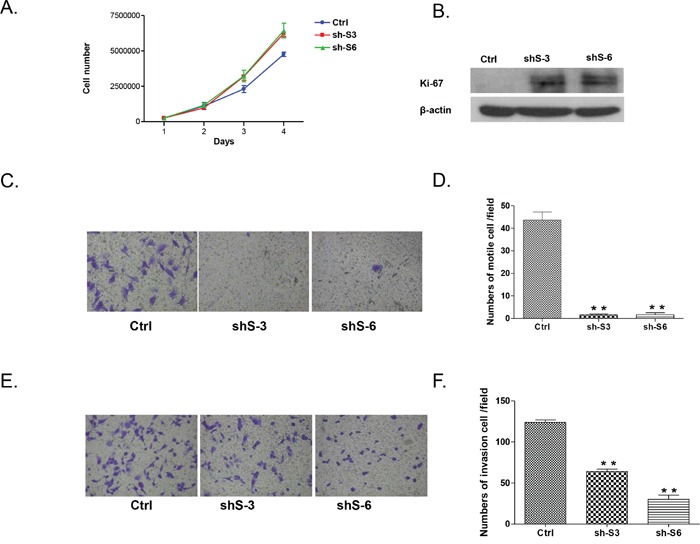
Effect of LY75 knockdown on SKOV3 cell proliferation migration and invasion **A.** Cell proliferation of LY75 knockdown clones sh-S3 and sh-S6 was compared to the control clone (Ctrl); **B.** Western blot analysis of the proliferation marker Ki-67 in LY75 knockdown clones sh-S3 and sh-S6 compared to the control clone. **C.** Cell migration of LY75 knockdown clones sh-S3 and sh-S6 was compared to the control clone (Ctrl). Migration was assessed using Boyden-chamber assay. Cells from the LY75 knockdown clones sh-S3 and sh-S6 and the Ctrl clone were seeded into the upper chambers in 0.1% FBS containing medium at a density of 2.5 × 10^4^ per well, and 600 μl of 1% FBS containing medium was placed in the lower chamber as a chemoattractant. After 24 h at 37°C in 5% CO_2_, the cells were fixed with cold methanol and stained with blue trypan solution. Migrated cells on the underside of the filter were photographed and counted by phase contrast microscopy. **E.** Cell invasion was assayed in a similar way, as the upper chambers were coated with Matrigel. Here, NIH3T3 conditioned medium was added in the lower chamber as a chemoattractant (see Methods for details). All experiments were performed in triplicate. For each experiment, cell number was calculated as the total count from 10 random fields per filter (at magnification × 40). The bar graphs in panels **D** and **F.** represent quantitative determinations of migration and invasion data obtained by selecting 10 random fields per filter under phase contrast microscopy and results are expressed as % change of the sh-S3 and sh-S6 clones over the Ctrl clone. Differences between shRNA-LY75 transfected and vehicle- transfected SKOV3 cells were determined by a Student's t-test; error bars denote mean ± SEM; *indicates statistical significance (p < 0.05).

Both LY75 knockdown clones sh-S3 and sh-S6 displayed increased proliferation rates compared to the control (Figure 4A), which was associated with overexpression of the proliferation marker Ki-67 (Figure 4B). However, LY75 suppression significantly inhibited both migration and invasion of SKOV3 cells (see Figures 4C–4F), which was also confirmed by a wound-healing assay (see Supplemental Figure 5). Thus, the *in vitro* invasiveness and motility of LY75 knockdown clones sh-S3 and sh-S6 inversely correlated with their proliferative potential, possibly due to the acquiring of the epithelial phenotype.

**Figure 5:**
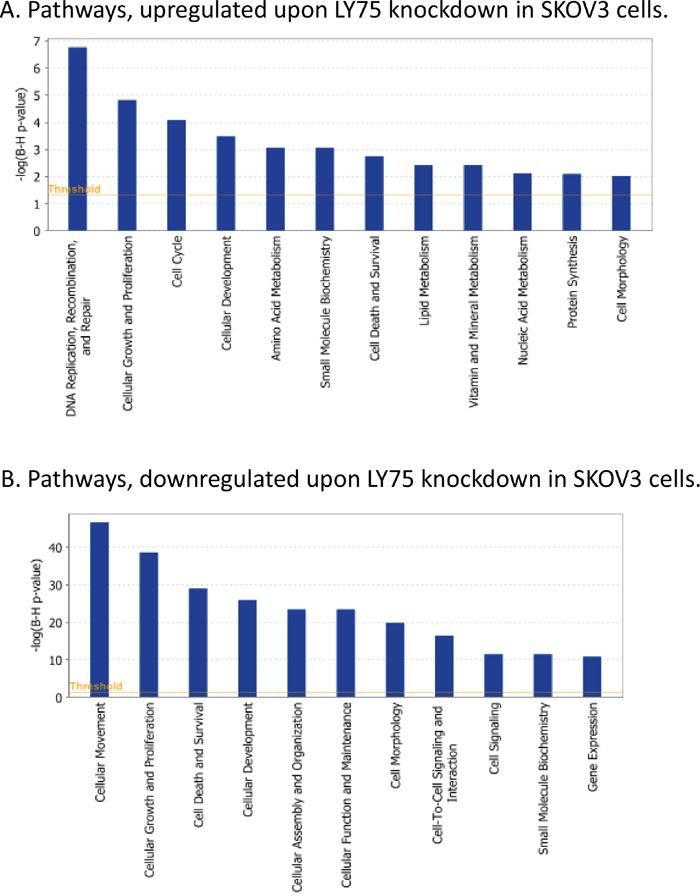
Functional analysis for a dataset of differentially expressed genes (≥ 2-fold) following LY75 suppression in SKOV3 cells **A.** Functional analysis of upregulated genes; **B.** Functional analysis of downregulated genes. Top functions that meet a Bonferroni-Holm multiple testing correction p-value of 0.05 are displayed.

**Figure 6:**
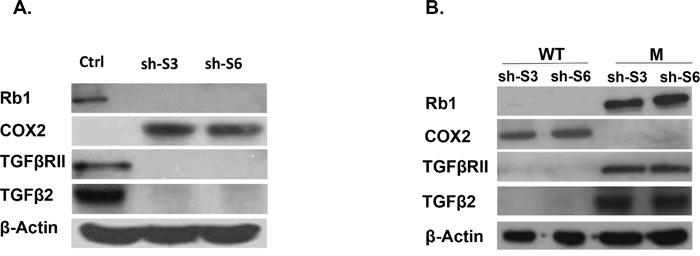
**A.** Western blot analysis of Rb1, TGFβ2, TGFβRII and COX2 protein expression in LY75 knockdown SKOV3 clones (sh-S3 and sh-S6) compared to the control cells (Ctrl). **B.** Western blot analysis of Rb1, TGFβ2, TGFβRII and COX2 protein expression in LY75 knockdown clones sh-S3 and sh-S6 upon ectopic expression of the wild-type (WT) or the mutated (M) LY75 gene.

Gene expression profiling sustained the major phenotype alterations in SKOV3 cells following LY75 suppression. Pathway and network analyses, generated through the use of the Ingenuity Pathway Analysis (IPA) software were indicative for predominant upregulation of functionally-related gene groups implicated in DNA replication recombination & repair, cell cycle, metabolism (including amino acid, lipid, vitamin, mineral and nucleic acid metabolism) and protein synthesis following LY75 knockdown (Figure 5A), while genes, functionally associated with cell movement, cellular assembly & organization, cell morphology, cell-to-cell signaling and interaction and cell signaling were mainly suppressed (Figure 5B). IPA canonical pathway analysis confirmed these findings, as the top upregulated canonical pathways were mostly related to lipid and amino-acids metabolism and cell cycle-mediated control of DNA replication, while significantly downregulated canonical pathways were predominantly associated with alterations in extracellular matrix (ECM) signaling and cell adhesion, complement activation and immune response modulation, including impaired DCs maturation and endocytosis signaling. More importantly, the EMT pathway and its major regulator – the TGF-β pathway [25] were among the top downregulated canonical pathways, which was evidenced by strong suppression of some major EMT modulators, such as TGF-β2 and TGFβRII (see Supplemental Table 2 and Figure 6A). Supplemental Figure 6 shows selected altered canonical pathways that were significantly dysregulated upon LY75 knockdown in SKOV3 cells. The restoration of the LY75 expression in both our LY75 knockdown clones (sh-S3 and sh-S6) was accompanied with the reestablishment of TGF-β2, and TGFβRII expression patterns, characteristic for the parental SKOV3 cells (Figure 6B). Supplemental Table 3 shows the complete list of the differentially expressed genes (≥2.0-fold at p value ≤0.05) following LY75 knockdown in SKOV3 cells; among these, the LY75 gene displayed significant medium suppression value (−16.76 fold; see Supplemental Table 3B), which essentially indicates for the complete LY75 knockout in both selected shRNA-LY75 clones.

Quite similar results were obtained when performing shRNA-mediated LY75 knockdown in the endometrioid EOC cell line TOV112, which also exhibits a mesenchymal-like phenotype. Indeed, using the shRNA construct #57364, we were able to generate the LY75 knockdown TOV112 clones sh-T5 and sh-T7 (see Supplemental Figure 3D and 3E), which displayed a typical epithelial morphology, accompanied with the overexpression of E-cadherin, EPCAM and EMP1 and the suppression of FN1, N-cadherin, SNAIL1 and TWIST1 (see Supplemental Figure 7). Likewise, TOV112 LY75 knockdown clones sh-T5 and sh-T7 exhibited increased proliferation and Ki-67 expression rates, while displaying lower migration and invasion capacities, when compared to control cells (see Supplemental Figure 8).

LY75 suppression in SKOV3 cells also led to robust overexpression of the PTGS2 (COX2) gene, representing a major EOC oncogene [26] (+70.92; see Supplemental Table 3), and the strong downregulation of the Rb1 tumor suppressor gene (−32.82 see Supplemental Table 3). The differential expression of both COX2 and Rb1 in the LY75 knockdown SKOV3 clones was further confirmed by Western blot analysis (Figure 6A), as the restoration of the LY75 expression re-established their initial expression pattern (Figure 6B). However, analogous COX2 and the Rb1 expression alterations were not observed following LY75 depletion in TOV112 cells, which suggests that the impact of LY75 silencing on these two genes might vary in EOC cell lines with different histological background.

### LY75 depletion in SKOV3 cells leads to increased tumor cell colonization and expansion in severe combined immunodeficiency (SCID) mice

**Figure 7:**
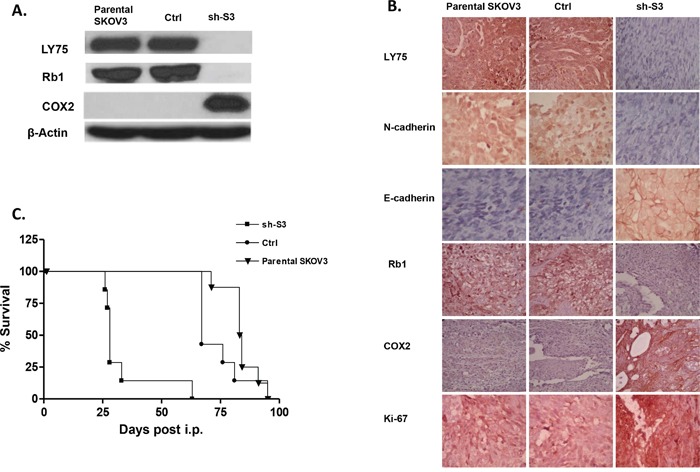
**A.** Western-blot analysis of LY75, Rb1 and COX2 protein expression in tumor tissues extracted from SCID mice, injected with the parental SKOV3 cells, the mock-transfected (Ctrl) cells, and the LY75 knockdown cells (sh-S3). **B.** Representative IHC images of LY75, E-cadherin, N-cadherin, Rb1, COX2 and Ki-67 expression in tumor tissues extracted from mice injected with the parental, Ctrl, and sh-S3 SKOV3 cells. **C.** Survival curves for mice injected with parental, Ctrl, and sh-S3 SKOV3 cells. The median survival of mice injected with the Ctrl cells (89 days, n = 8) was similar to the parental SKOV3 cells (86 days, n = 7). Survival of mice injected with the LY75 knockdown cells was significantly shorter than the vector control (40 days; p<0.0001, Log rank test).

EOC spreads by intraperitoneal (IP) sloughing, lymphatic invasion, and hematogenous dissemination [27]. IP dissemination is the most common; after malignant cells have evaded from the ovarian capsule, they are shed from the tumor surface into the peritoneal cavity where they follow normal routes of peritoneal fluid [28]. Hence, IP injection of cancer cells in animal models can accurately model advanced disease, as EOC metastases frequently appear disseminated throughout the peritoneum [29]. We used a similar *in vivo* approach; thus, intact SKOV3 cells, as well as control and shRNA-LY75 transduced SKOV3 cells (clone sh-S3) were IP injected in SCID mice (n=7-8 per experimental group). The LY75 suppression in tumors of mice injected with the LY75-knockdown cells was later confirmed by Western blot and IHC staining (Figure 7A and 7B). We found that LY75 knockdown had a major impact on survival. While the median survival of mice injected with the vector-control cells (89 days, n=6) was similar to the parental SKOV3 cells (86 days, n=7), the survival of mice injected with LY75 knockdown cells was significantly shorter than the vector-control (40 days; (p<0.0001; Logrank Test) (Figure 7C). Moreover, the total tumour burden in mice injected with parental (aver. 1.0 g) and control (aver. 1.1 g) SKOV3 cells was significantly lower than in mice injected with LY75 knockdown cells (aver. 5.6 g; see Supplemental Figure 9).

Consistent with our previous data *in vitro*, tumor specimens derived from sh-S3 cells stained strongly for E-cadherin when compared with parental SKOV3 and control derived xenografts, whereas N-cadherin was only present following the injection of parental and control cells (Figure 7B). These data sustain *in vivo* our findings for the implication of LY75 in controlling the EMT. The proliferation marker Ki-67 was also induced in tumor tissues derived from mice injected with the LY75-knockdown cells, in agreement with our *in vitro* functional analyses (Figure 7B). Finally, the strong induction of COX2 expression and the Rb1 suppression following LY75 knockdown in SKOV3 cells was also confirmed *in vivo* by Western blot and IHC (see Figures 7A and 7B).

### LY75 inactivation directs EMT in EOC cell lines with epithelial phenotype, associated with reduced capacity for *in vivo* EOC cell colonization

**Figure 8:**
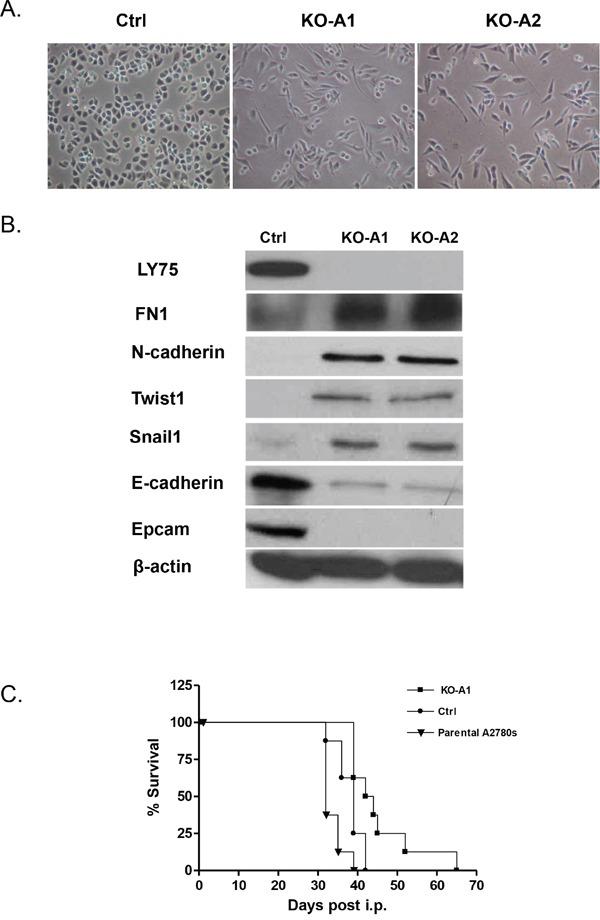
**A.** Representative phase-contrast images of A2780s control and LY75 knockout clones (KO-A1 and KO-A2). **B.** Western blot analysis of the expression of different EMT markers in the control and the LY75 knockout A2780s clones. **C.** Survival curves for mice injected with parental, Ctrl, and LY75 knockout (clone KO-A1) A2780s cells. The median survival of mice injected with the LY75 knockout cells (43 days) was significantly prolonged compared to mice injected with the parental (34 days; p=0.0065, Log rank test) or control cells (37 days; p=0.0153, Log rank test).

**Figure 9:**
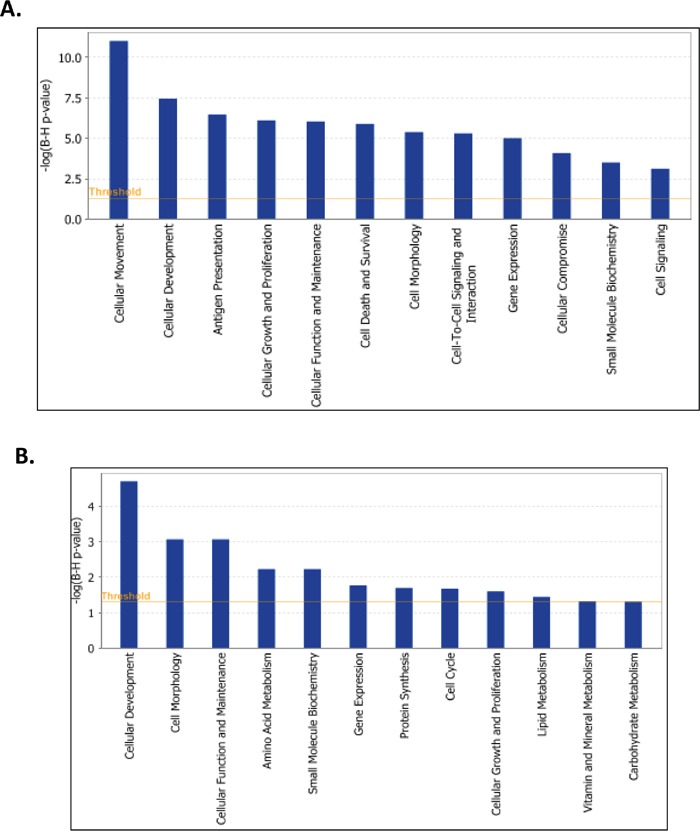
Functional analysis for a dataset of differentially expressed genes (≥ 2-fold) following LY75 suppression in A2780s cells **A.** Functional analysis of upregulated genes; **B.** Functional analysis of downregulated genes. Top functions that meet a Bonferroni-Holm multiple testing correction p-value of 0.05 are displayed.

Since the LY75 gene displayed strong endogenous expression in all EOC cell lines tested (see Supplemental Figure 3A), we decided to also verify the effect of LY75 suppression in EOC cell lines with typical epithelial morphology. Quite surprisingly, CRISPR/Cas9-mediated LY75 gene knockout directed EMT in A2780s cells (Figure 8A), associated with the acquirement of spindle-like cellular phenotype and the expression of N-cadherin, TWIST1, FN1 and SNAIL1 compared to the control, while the expression E-cadherin and EPCAM was strongly down-regulated (Figure 8B). Gene expression profiling confirmed these findings, as identical or similar functional networks and canonical pathways found to be dysregulated upon LY75 knockdown in SKOV3 cells, were reversibly altered following LY75 depletion in A2780s cells. Indeed, functionally-related gene groups implicated in cell movement, cellular assembly & organization, cell morphology, cell-to-cell signaling and interaction and cell signaling were predominantly upregulated in LY75 knockout A2780s clones, while genes, functionally associated with control of metabolism (including amino acid, lipid, vitamin, mineral and carbohydrate metabolism) and protein synthesis were mainly suppressed (see Figure 9). Similarly, the top upregulated canonical pathways were associated with induction of EMT, ECM signaling and cell adhesion, complement activation and immune response modulation, including DCs maturation and endocytosis signaling, while significantly downregulated canonical pathways were mostly related to altered lipid & amino acids metabolism (see Supplemental Figure 10). Supplemental Table 4 shows the complete list of the differentially expressed genes (≥2.0-fold at p value ≤0.05) following LY75 knockout in A2780s cells. Similar results were obtained upon LY75 suppression in the EOC cell line OV2008 (data not shown). Moreover, LY75 knockout in A2780s cells led to significantly prolonged survival *in vivo* upon IP injection in SCID mice, compared to parental (p = 0.0001; Logrank Test), or control A2780s cells (p=0.0153; Logrank Test) (Figure 8C). The LY75 suppression in tumors of mice injected with the LY75-knockout cells was later confirmed by IHC (Supplemental Figure 11). These data suggest that the LY75 is implicated in modulating both EMT/MET in EOC cells, depending on the EOC cellular context/phenotype.

## DISCUSSION

The mechanisms of EOC tumorigenesis, progression and dissemination have not been yet fully clarified. Using an epigenomics technology, we have recently identified LY75 gene as a novel potentially hypomethylated target in advanced EOC [3]. In the present study, we demonstrated that LY75 is overexpressed exclusively in HG serous ovarian tumors, as compared to LMP tumors and normal ovarian tissues. Applying an independent (BSP) approach, we also confirmed the hypomethylation status of the putative LY75 gene promoter region in HG EOC tumors, suggestive for epigenetic regulation of LY75 expression in late disease. Similarly, a recent *in silico* analysis based on the NCBI GEO database was also indicative for the significant overexpression of LY75 in serous EOC compared to normal ovary [30]. Moreover, by using the cBioPortal online analytical tool [31, 32] which includes the TCGA ovarian serous cystadenocarcinoma database [33], we also found that LY75 displays the highest amplification rate in serous EOC, compared to all other cancer types.

Few reports suggest for possible role of LY75 in malignant diseases; thus, LY75 has been reported as suppressed in colorectal [34] and breast cancer [35], but universally expressed in both bladder carcinomas and normal urothelium [36] and induced in thymoma [37]. The implication of LY75 in EOC tumorigenesis is poorly understood, although it has been shown that depletion of LY75-positive DCs in mice with established EOC impairs cancer progression [38]. A recent study was also suggestive for a potential oncogenic role for LY75 in EOC, by demonstrating that the IL6 oncogene and its receptor IL6Ra control LY75 overexpression in EOC, which contributes to EOC dissemination [30]. A role of LY75 in mediating cell adhesion was also postulated, as LY75 upregulation resulted in enhanced adherence of EOC cells to the omentum in *ex vivo* and *in vivo* EOC animal models [30].

Here we report for the first time a new role of LY75 gene in controlling EOC cellular phenotype and corresponding metastatic potential. We demonstrated that LY75 depletion directs MET in EOC cell lines with mesenchymal-like morphology (SKOV3 and TOV112), associated with the induction of the expression of the epithelial markers E-cadherin, EPCAM and EMP1 and loss of expression of the mesenchymal markers N-cadherin, TWIST1, FN1 and SNAIL1. Moreover, re-expression of the LY75 gene in LY75 knockdown SKOV3 clones completely restored the initial mesenchymal phenotype and re-established the pattern of epithelial and mesenchymal markers' expression, characteristic for the parental SKOV3 cells. LY75 knockdown in SKOV3 cells resulted in predominant upregulation of functionally-related pathways implicated in cell proliferation and metabolism, while pathways associated with alterations in ECM signaling and cell adhesion, complement activation and immune response were mostly suppressed. Interestingly, the acquisition of epithelial phenotype upon LY75 knockdown was associated with reducing the migration and invasion of EOC cells *in vitro*; however, the loss of LY75 expression significantly enhanced tumor cell colonization and expansion *in vivo* in IP xenograft mouse model.

Quite unexpectedly, LY75 suppression had the opposite effect in the EOC cell lines A2780s and OV2008, both displaying epithelial phenotype. Moreover, LY75 depletion in A2780s cells was associated with reduced capacity for *in vivo* EOC cell colonization, which also confirms the higher potential of epithelial cancer cells for distant colonization and metastatic tumor formation. LY75 knockout triggered EMT in these cell lines, as similar or identical functional and canonical pathways were reversely regulated, when compared to SKOV3 LY75 knockdown cells (see Supplemental Figures 6 and 11).

To our knowledge, this is the first report of a gene displaying pleiotropic effects in sustaining the cellular phenotype of EOC cells and points to novel functions of this receptor in modulating EOC dissemination. Evidently, such a “gatekeeper” function of LY75 might be due to its differential association with distinct co-factors which, depending on EOC cellular phenotype, could modulate either the induction or the suppression of similar/identical functional and canonical pathways that control EOC cell morphology and metastatic potential.

Our data also suggest that similar factors/pathways that modulate EMT could be implicated in DCs maturation. Indeed, maturation of DCs is accompanied with significant changes in DCs cellular morphology [39], and a number of cytokines (including TGFβ, IL-10, IL-18, IFNα, IFNγ) that are modulated by LY75 suppression in EOC cells, are shown to be also involved in DCs maturation [40]. Unlike the other members of the mannose receptor family, LY75 is massively up-regulated on DCs maturation, due both to *de novo* synthesis and increased shuttling to the cell surface, which is also associated with a loss of LY75 endocytic activity on mature DCs [41]. As mature DCs interact with secondary lymphoid organs, LY75 could also mediate interactions with ECM proteins, endothelium, and/or enhance DCs/T-cell interactions in lymph nodes [41].

Metastasis is a complex multistep process in the progression of cancer, causing approximately 90% of all human cancer mortalities [42]. To colonize a distant secondary site, cancer cells undergo EMT characterized by the suppression of epithelial markers E-cadherin and EPCAM and acquisition of migratory capacity pivotal for invasion and metastasis [43, 44]. Although EMT is clearly important to tumor progression, it is inconsistent with the observation that metastatic lesions mostly exhibit epithelial phenotypes [45], thus suggesting that MET is critical to the latter stages of metastasis. It has been lately suggested that circulating tumor cells can form clusters containing cells with both epithelial/mesenchymal or partial/intermediate EMT phenotype, which display higher tumor-initiating potential and increased drug resistance compared to either epithelial or mesenchymal cells alone [46]. Moreover, recent findings provided evidence that epithelial tumor cells do not need EMT to form metastases, but that EMT rather mediates cancer-cell survival by contributing to chemoresistance [47].

Our data are in agreement with previous findings, demonstrating that cancer cells with epithelial phenotype display reduced migration and invasion capacity *in vitro*, but exhibit superior capacity to colonize secondary sites *in vivo*, when compared to mesenchymal-like cancer cells [42, 48–55]. Indeed, overexpression of different epithelial markers was observed in the metastatic sites of ovarian [56], breast [57–60], colorectal [61, 62], prostate [63, 64], lung [65, 66], and gastric cancer [67], which is a strong indication that the cancer cells in the primary tumor and their metastatic lesions share a similar epithelial nature. Interestingly, the “Role of Oct4 in Mammalian Embryonic Stem Cell Pluripotency” canonical pathway was among the pathways, significantly upregulated upon LY75 knockdown in SKOV3 cells (see Supplemental Figure 6A), which supports literature data underlying the superior capability of cancer cells bearing the epithelial phenotype to maintain ES cells-like features that are essential for self-renewal, pluripotency, and enhanced invasiveness [50]. Accordingly, our results raise concern on the use of LY75-targeted vaccines for dendritic-cell EOC immunotherapy, due to the possible side effects that can be generated following LY75 blockade in EOC cells, associated with alterations in cellular phenotype and metastatic potential.

In conclusion, we have shown that the mannose receptor LY75 is significantly overexpressed in HG serous EOC tumors compared to LMP tumors and normal ovarian tissues, as epigenetic mechanisms might modulate LY75 overexpression in advanced disease. More importantly, we report for a novel function of this endocytic receptor in regulating EMT and in maintaining EOC cellular phenotype and capacity to colonize distant sites. Our data also support previous findings concerning the superior metastatic potential of cancer cells bearing epithelial phenotype as compared to cancer cells with mesenchymal-like morphology. Further studies are warranted to better understand the molecular mechanisms of LY75-mediated control on EOC cell morphology and metastatic potential, and possibly, its analogous functions in sustaining cellular phenotype and invasive capacities of tumor cells derived from other cancer types.

## MARERIALS AND METHODS

### Patients and tissue specimens

Snap frozen and formalin-fixed paraffin-embedded (FFPE) tissues of 117 serous EOC tumors were provided by the Banque de tissus et de données of the Réseau de recherche sur le cancer of the Fonds de recherche du Québec - Santé at the Hotel-Dieu de Quebec Hospital, Quebec, Canada, which is affiliated with the Canadian Tumor Repository Network. These clinical specimens included 13 borderline, or low-malignant potential (LMP) tumors, 52 high-grade adenocarcinomas and 52 omental metastases. None of the patients received chemotherapy before surgery (see Supplemental Table 1 for detailed clinicopathological characteristics). All tumors were histologically classified according to the criteria defined by the World Health Organization [68]. The CT treatment was completed for all patients and the response to treatment was known. Disease progression was evaluated following the guidelines of the Gynecology Cancer Intergroup [68]. Progression free survival (PFS) was defined as the time from surgery to the first observation of disease progression, recurrence or death. Thirteen normal ovarian samples and 13 normal uterine smooth muscle samples were derived from women subjected to hysterectomy with oophorectomy due to non-ovarian pathologies. This study was approved by the Clinical Research Ethics Committee of the Hotel-Dieu de Quebec Hospital and patients gave written consent for tissue collection and analyses.

### Cell cultures

The EOC cell lines OVCAR3 and SKOV3 were purchased from American Tissue Type Collection (Manassas, VA); OV-90, OV2008, TOV-112 and TOV-21 cell lines were a kind gift from Dr. Anne-Marie Mes-Masson (Université de Montréal), while A2780s and A2780cp cell lines were a kind gift from Dr. Benjamin Tsang (University of Ottawa). The TOV-155 cell line was recently established in our lab as spontaneously immortalized EOC cell line from poorly differentiated serous EOC tumor and the detailed characterization of this cell line will be reported elsewhere. The cell lines were passed in different culture media supplemented with 10% fetal bovine serum, as previously described [7].

### Bisulfite sequencing PCR (BSP) analysis

BSP analysis was performed, as previously described. Briefly, genomic DNAs from EOC tumors and control ovarian tissues were isolated using the Qiagen DNeasy Blood and Tissue Kit. Bisulfite modification of genomic DNAs was done using the Methyl Detector kit (Active Motif, Carlsbad, CA). For BSP, a 348-bp fragment was amplified using primer pairs specific for bisulfite-modified sequences but not harboring CpGs, located at nt + 304 (GATTTTGAGAGGGAGGGT) to nt + 619 (ATCAAAAAAATAAACCCAACTCT) downstream of the LY75 transcription start (ATG) codon. BSP primer selection was performed using the Methyl Primer Express Software v1.0 (Applied Biosystems). PCR was done for 30 cycles (94°C, 45 s; 60°C, 45 s; 72°C, 45 s). PCR products were sent for dideoxy-sequencing analysis at the Genomics Analysis Platform at Laval University (http://www.bioinfo.ulaval.ca/seq/en/).

### Tissue microarrays (TMAs) construction and immunohistochemistry (IHC)

TMAs were constructed, as previously described [6]. Briefly, one representative block of each ovarian tumor and normal ovarian tissue was selected for the preparation of the tissue arrays. Three 0.6 mm cores of tumor were taken from each tumor block and placed, 0.4 mm apart, on a recipient paraffin block using a commercial tissue arrayer (Beecher Instruments, Sun Prairie, WI). The cores were randomly placed on one of two recipient blocks to avoid IHC evaluation biases. Four micron thick sections were cut for the hematoxylin-eosin (HE) staining and IHC analyses. IHC was performed, as previously described [6]. Briefly, 4 mm tissue sections were deparaffinized and then heated in an autoclave for 12 min to retrieve the antigenicity before blocking with endogenous peroxidase. Following treatment with 3% H_2_O_2_ for 10 min to quench the endogenous peroxidise activity, sections were incubated with anti-LY75 antibody (1:100 dilution) (LSbioSpan) at room temperature for 2 hours. Sections were then incubated with a biotinylated secondary antibody (Dako, Carpinteria, CA) and then exposed to a streptavidin complex (Dako, Carpinteria, CA). Complete reaction was revealed by 3,3′-diaminobenzidine and slides were counterstained with hematoxylin. LY75 protein expression was assessed by semi-quantitative scoring of the intensity of staining and recorded as absent (0), weak (1+), moderate (2+) or strong (3+). The relationship between LY75 expression in serous ovarian carcinomas and normal ovarian tissues was evaluated by the Mann-Whitney test. A significant association was considered when p-value was below 0.05. A Kaplan Meier curve and the log-rank test were performed based on PFS values to test the effect of the intensity of LY75 (3, 2 versus 0, 1) on disease progression.

### Short hairpin RNA (shRNA) - mediated LY75 knockdown in EOC cells

The shRNA-mediated LY75 knockdown in EOC cells was done, as previously described [5, 7]. Briefly, two LY75 shRNAs cloned into the pLKO.1-puro vector (targeting the LY75 mRNA sequences 5′-GCCCUAAUACUCAACCUCCAA-3′ and 5′-UCCCGTCUUACAUAUUCAUCAA-3′) were retrieved from the Sigma Mission TRC human 1.5 shRNA library (clone numbers TRCN0000057363 and TRCN0000057364). Viral supernatants were generated by transfecting 293T cells with the shRNA constructs and the packaging vectors psPAX2 and pMD2.G (Addgene, Cambridge, MA). The high-titer lentiviral supernatants in the presence of 8 mg/ml polybrene were used to infect SKOV3 and TOV112 cells. Two days later, infected cells were treated with puromycin (0.5 mg/ml) for the selection of stably-transduced clones. The pLKO.1-puro vector encoding a scramble sequence not matching any mammalian sequence was used for the generation of mock-transduced (control) clones. Stable clones with inhibited LY75 expression were evaluated and validated by quantitative RT-PCR and Western blot.

### CRISPR/Cas9 mediated LY75 knockout in EOC cells

CRISPR/Cas9 mediated LY75 knockout in EOC cells (SKOV3 and A2780s) was done according to the manufacturer's guidelines (Santa Cruz Biotechnology, Inc). Briefly, 1.5 × 10^5^ cells were plated onto 6 × 30-mm well plates and allowed to grow to 70% confluence. Five microliters of UltraCruz Transfection Reagent (sc-395739) were added to 20 μg of DEC-205 CRISPR/Cas9 KO Plasmid (sc-417220) and 20 μg DEC-205 HDR Plasmid (sc-417220-HDR). The complexes were incubated at room temperature for 20 min and then overlaid onto the cells. The plates were then incubated at 37°C, 5% CO_2_ for 48 h. Stably transfected clones were selected by adding puromycin (0.5 mg/ml). Selected clones were transfected with Cre vector (sc-418923) for the removal of genetic material flanked by LoxP sites. The control CRISPR/Cas9 Plasmid (sc-418922) contained a non-targeting 20 nt scramble guide RNA (gRNA) designed as a negative control. Thus, the Cas9/gRNA complex does not recognize any DNA sequence and will not bind or cleave genomic DNA.

### Ectopic LY75 re-expression in LY75-knockdown SKOV3 cells

For LY75 ectopic expression, constructs containing wild type (WT) or mutated (M) cDNA of the human LY75 gene cloned in the pcDNA3.1+-DYK eukaryotic expression vector, were purchased from GenScript (Piscataway, NJ, USA). Transfection with ExGen 500 (Fermentas Canada Inc., Burlington ON) was carried out according to the manufacturer's guidelines. Briefly, 1 × 10^5^ SKOV3 cells (control and LY75-knockdown) were plated onto 6 × 30-mm well plates and allowed to grow to 70% confluence. Ten microliters of ExGen 500 were added to 2 μg of plasmid DNA dissolved in 190 μl of 150 mM NaCl. The complexes were incubated at room temperature for 10 min and then overlaid onto the cells in 1.8 ml medium. The plates were then incubated at 37°C, 5% CO_2_ for 48 h. Stably-transfected clones were selected by adding neomycin (30 μg/ml) and further cultivation for about 2 weeks. Cells were also mock-transfected with the pCMV6 vector, and stably transfected clones were isolated as controls. The LY75 expression levels of selected LY75-overexpressing and control clones were confirmed by Western blot.

### Western blot analysis

Western blot analysis was performed as previously described [4–7]. Briefly, protein lysates were prepared by re-suspending cell pellets in Laemmli sample buffer containing 5% b-mercaptoethanol. Protein lysates were separated by 6 to 15% Tris-glycine gel electrophoresis and transferred onto a polyvinylidene difluoride membrane using a semi-dry apparatus (Bio-Rad Laboratories, Hercules, CA). The membrane was blocked with 5% nonfat dry milk in TBST (20 mmol/L Tris-HCl, 0.5 M NaCl, and 0.1% Tween 20), incubated with the anti-LY75 and EMP1 antibodies (LSbioSpan), anti-Snail1, FN1, E-cadherin, EPCAM, Ki-67, TGFβ2, TGFβRII, Cox-2, Rb and β-actin antibodies (Santa Cruz Biotechnology) and anti-Twist1 and N-cadherin antibodies (Abcam) at 4°C overnight. After 3 washes with TBST (20 mmol/L Tris-HCl, 0.5 M NaCl, and 0.1% Tween 20) at room temperature, the membrane was incubated with horseradish peroxidase-conjugated secondary antibody, then treated with ECL solution (Thermo Fisher Scientific, Waltham, MA) and detected on blue sensitive autoradiography film (Marsh Bio Products, Rochester, NY).

### Proliferation assay

Cell proliferation was evaluated by Malassez cell counting. Briefly, cells were cultured at a density of 0.25 × 10^6^/plate. Cells were counted after 48, 72 and 96 h using trypan bleu staining. Assays were performed in triplicate. Differences between shRNA-LY75 knockout clones (sh-S3 and sh-S6, or sh-T5 and sh-T7) and the corresponding control (ctrl) clones were determined by the one-way ANOVA analysis, where p < 0.05 was considered significant.

### Cell migration and invasion assays

Cell migration and invasion assay were performed, as previously described [4–7]. Briefly, LY75 shRNA-transduced and control (scramble shRNA) cells were seeded into the upper inserts of Boyden chambers (Costar, Cambridge, MA) in 0.1% FBS containing medium at a density of 2.5 × 10^4^ per well; then, 600 μl of 1% FBS containing medium was added in the lower chamber as a chemoattractant. After 24 h at 37°C in 5% CO_2_, the cells were fixed with cold methanol and stained with trypan blue solution. Cells on the upper surface of the filter were removed with cotton buds. Migrated cells on the underside of the filter were photographed and counted by phase contrast microscopy, by selecting 10 random fields per filter (at magnification × 40). The experiments were performed in triplicate.

Cell invasion was assayed in a similar way, as the 5-mm pore polycarbonate filters were coated with 40 μl of Matrigel™ at concentration of 0.5 mg/ml (BD Biosciences, Franklin Lakes, NJ). Here, 600 μl of NIH3T3 conditioned medium was added in the lower chamber as a chemoattractant. Differences between shRNA-LY75 clones (sh-S3 and sh-S6, or sh-T5 and sh-T7) and the corresponding control (ctrl) clones were determined by a Student's t-test, where p < 0.05 was considered significant.

### Gene expression profiling and data analysis

Gene expression analysis was carried out as previously described [4–7]. Briefly, total RNA was extracted from the selected shRNA-LY75 SKOV3 knockdown clones (sh-S3 and sh-S6), and the A2780s LY75 knockout clones (KO-A1 and KO-A2), and the corresponding controls. The quality of the RNA samples was examined by capillary electrophoresis using the Agilent 2100 Bioanalyzer (Agilent, Palo Alto, CA). Fluorescently labeled cRNA targets were generated from 0.5 mg of total RNA from each corresponding SKOV3 cell clone, using the Fluorescent Linear Amplification Kit (Agilent) and 10.0 mM Cyanine 3- or 5-labeled CTP (PerkinElmer, Boston, MA), and following user's manual. Cyanine labeled cRNA from the LY75 knockdown/knockout SKOV3/A2780s clones was mixed with the same amount of reverse-color cyanine-labeled cRNA from the corresponding control clones and hybridized on the Agilent Whole Human Genome microarrays, containing 44,000 genes. Array hybridization, washing and scanning were performed as previously described [4–7]. The subsequent data analyses were performed using Partek Genomics Suite Version 6.4 (Partek, St Louis, MO, USA). Differentially expressed genes were selected at ≥2-fold difference between LY knockdown clones and control, and p< 0.05. Principal component analysis was conducted with Partek default settings. Network analysis of the microarray data was completed using the IPA software. The microarray data have been deposited to GEO database with accession number GSE74711.

### Quantitative PCR (qPCR)

Quantitative PCR was performed as previously described [6]. Briefly, total RNA was extracted by the RNeasy Plus Mini Kit (QIAGEN) and cDNA was obtained by the qScript™ cDNA SuperMix (Quanta BioSciences, Inc.). Primers were designed for these loci with the sequences freely available from the Entrez Nucleotide database and the Primer3 algorithm for primer design (http://www-genome.wi.mit.edu/cgi-bin/primer/primer3_www.cgi). PerfeCTa^®^ SYBR^®^ Green FastMix^®^ (Quanta BioSciences, Inc.) was used according to manufacturer's instructions. PCR reactions were performed on Rotor-Gene RG-3000 Real Time PCR System (Qiagen), with 18S ribosomal RNA used as endogenous control. PCR volume was 20 μl and conditions were as follow: initial cycle 50°C, 2 min, 95°C, 15 min; 45 cycles at 95°C, 20 s, 60°C, 20 s and 72°C, 20 s; final cycle 72°C, 30 s. Data were analyzed by the Rotor-Gene software using the comparative ΔΔCt method. The relative copy number was calculated based on the target gene/18S RNA ratio.

### Immunofluorescence

Cells were plated on poly-D-lysine coated slides (Sigma) and were fixed in 4% paraformaldehyde and permeabilized with Triton 1% X-100. After blocking, cells were incubated with primary antibodies, anti–E-cadherin (Abcam, 1:50) and anti-N-cadherin (Abcam, 1:50), subsequently with Rhodamine-linked goat-anti-mouse IgG1 (Santa Cruz, 1:50) and with Alexa Fluor 488–labeled secondary antibody (Life technologies, 1:500) respectively, and finally stained with 4′,6-diamidino-2-phenylindole (DAPI). Images were captured using a Zeiss LSM 510 confocal microscope system.

### Peritoneal tumor formation in mice

Intact SKOV3 cells, as well as cells from the SKOV3 LY75 knockdown sh-S3 clone and the SKOV3 ctrl clone (1 × 10^7^ cells in 500 μL of PBS), were IP injected into 8 × 8 week old CB17 SCID female mice (CB17/Icr-Prkdcscid/IcrIcoCrl strain code 236, Charles River) using a 25G5/8 needle, as previously described [7]. Mice were monitored daily by staff blinded to the cell type injected and euthanized when they reached a loss of wellness endpoint that was most often respiratory distress associated with ascites accumulation. The animals had free access to food and water and experiments were done in accordance with the Canadian Council on Animal Care's Guidelines for the Care and Use of Animals. Protocols were approved by the University of Ottawa Animal Care Committee.

## SUPPLEMENTARY FIGURES AND TABLES








