# Supplementary material for: The mannose receptor LY75 (DEC205/CD205) modulates cellular phenotype and metastatic potential of ovarian cancer cells
Source: Oncotarget. 2016 Feb 9;7(12):14125–42. doi: 10.18632/oncotarget.7288 (PMC4924702; doi:10.18632/oncotarget.7288)
Supplement: Supplementary file 1 [file oncotarget-07-14125-s001.pdf]

SUPPLEMENTARY FIGURES AND TABLES

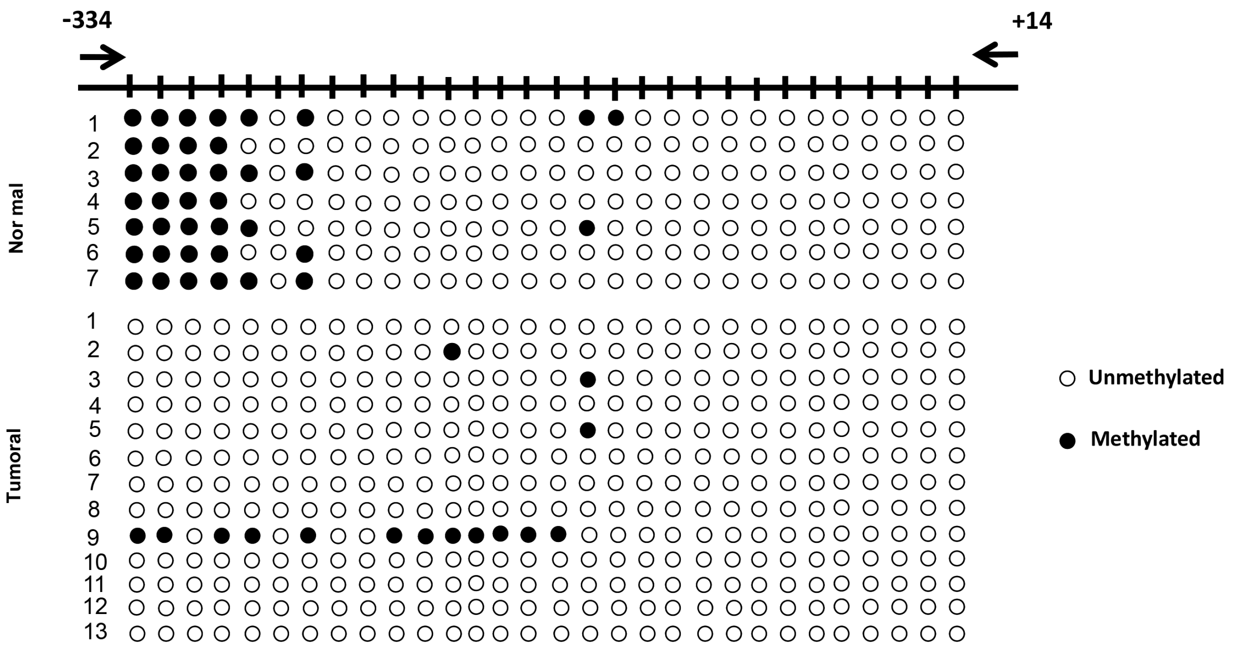

Supplementary Figure S1: BSP analysis of the methylation status of LY75 in HG serous EOC tumors compared to normal ovarian tissue. Filled circles represent methylated CpGs and open circles represent unmethylated CpGs. CpG plot of the analyzed region is also presented (CpGs are displayed with vertical marks). The indicated positions on the CpG plot represent the number of nucleotides stretching up- or down-stream of the start (ATG) codon of the LY75 gene and covering its putative promoter region.

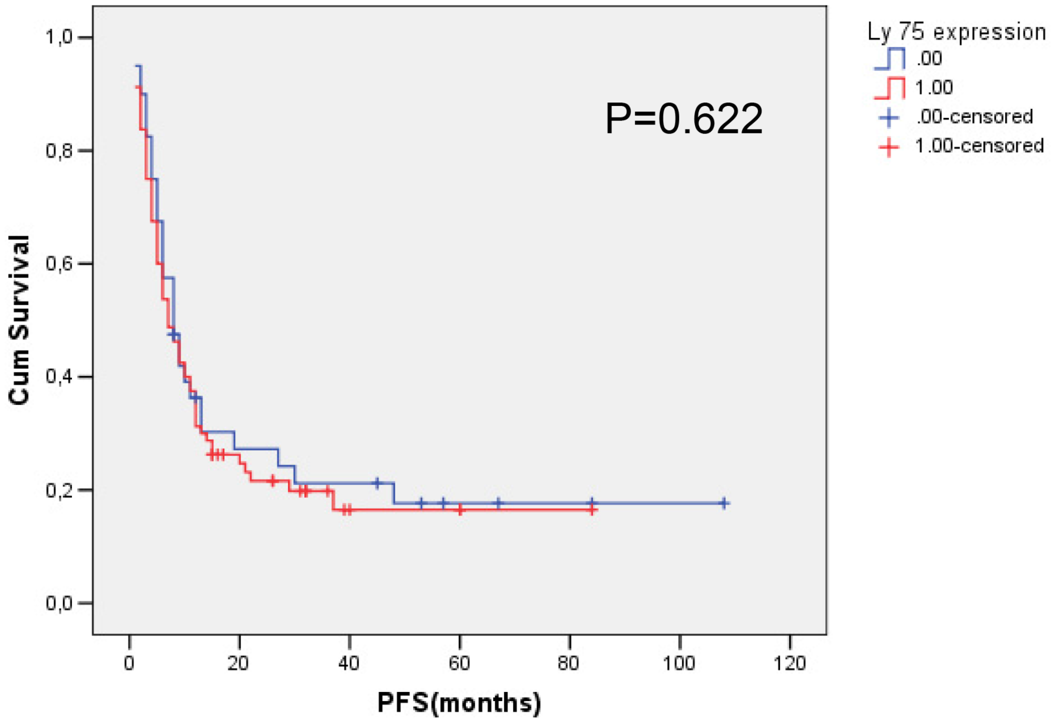

Supplementary Figure S2: Kaplan-Meier curve for progression free survival according to the level of LY75 IHC intensity in tumor samples of 103 serous EOC patients.

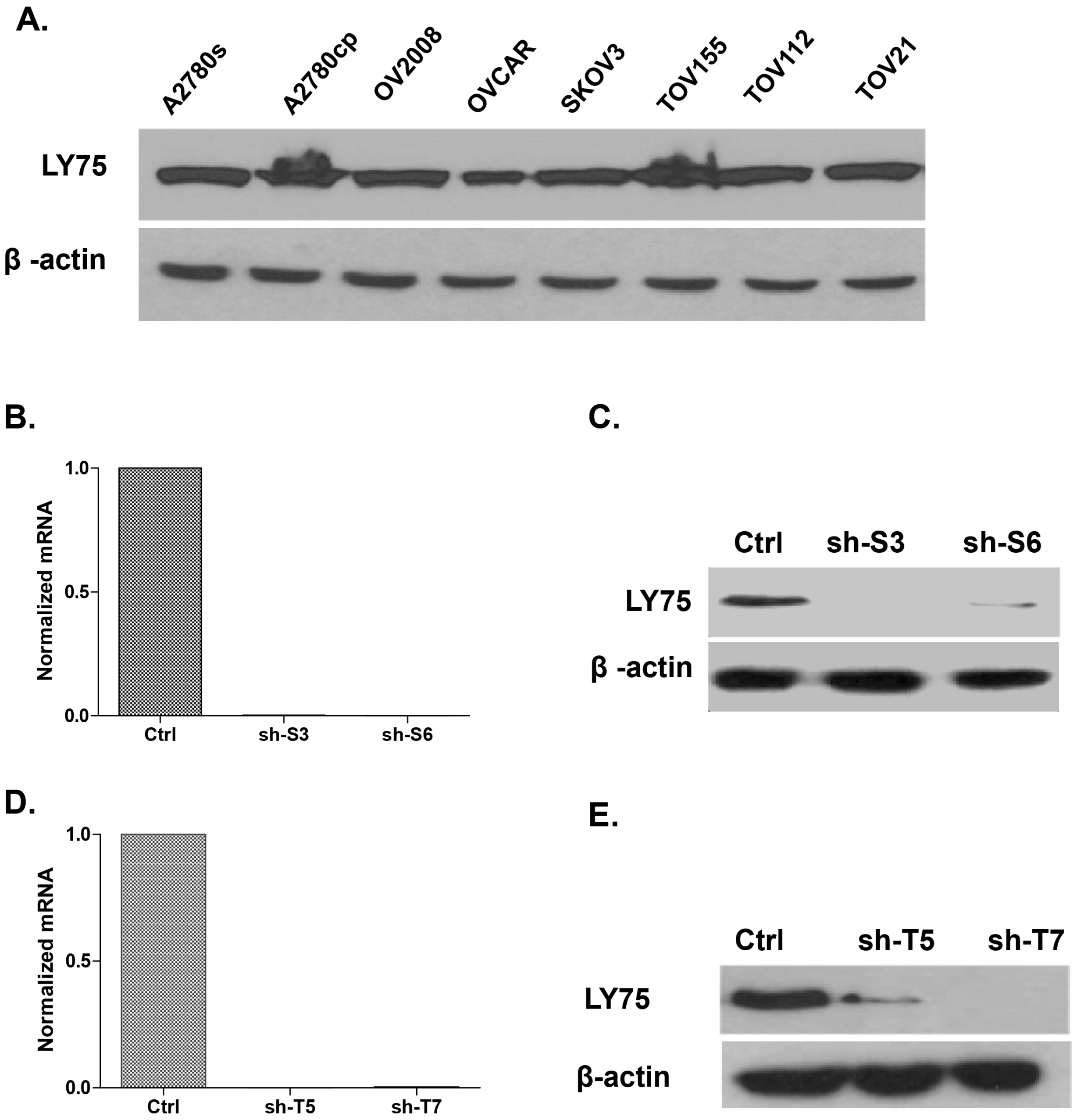

Supplementary Figure S3: shRNA-mediated LY75 knockdown in the EOC cell lines SKOV3 and TOV112. A. Western-blot analysis of endogenous LY75 protein expression in different EOC cell lines. **B.** Quantitative PCR (qPCR) analysis of LY75 mRNA expression levels in the shRNA-LY75 SKOV3 clones sh-S3 and sh-S6, compared to the mock-transfected control clone (Ctrl). **C.** Western-blot analysis of LY75 protein expression in the shRNA-LY75 SKOV3 clones sh-S3 and sh-S6, compared to the control clone (Ctrl). *β*-actin was used as a loading control. **D**. qPCR analysis of LY75 mRNA expression levels in the shRNA-LY75 TOV112 clones sh-T5 and sh-T7, compared to the mock-transfected control clone (Ctrl). **E.** Western-blot analysis of LY75 protein expression in the shRNA-LY75 TOV112 clones sh-T5 and sh-T7, compared to the control clone (Ctrl). *β*-actin was used as a loading control.

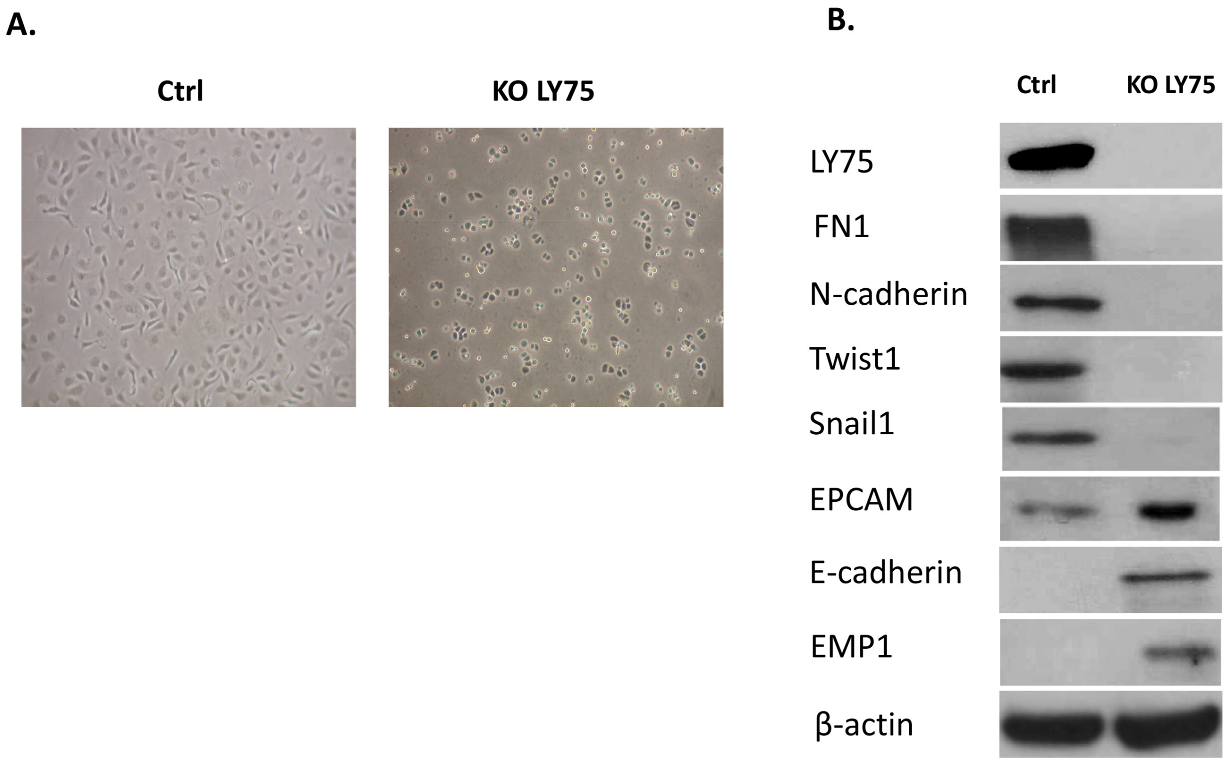

Supplementary Figure S4: CRISPR/Cas9-mediated LY75 suppression directs MET in SKOV3 cells. A. Representative phase contrast images of SKOV3 control and LY75 knockout clone. **B.** Western blot analysis of the expression of LY75 and different EMT (epithelial and mesenchymal) markers in the control and the LY75 knockout SKOV3 clone.

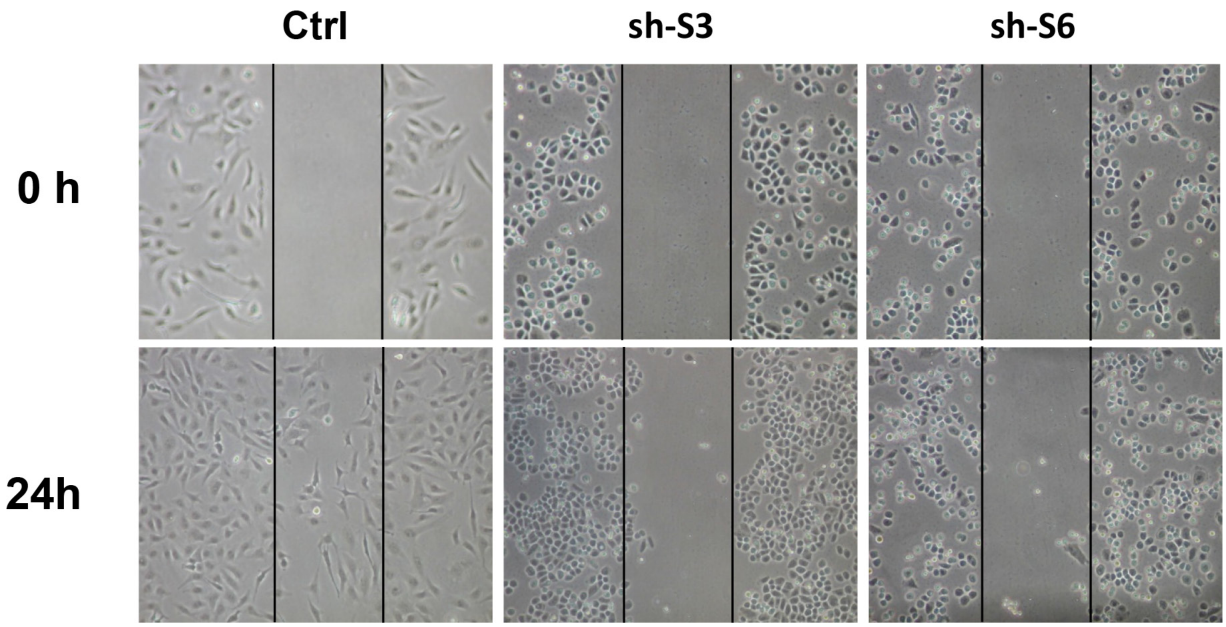

Supplementary Figure S5: Effect of shRNA-mediated LY75 knockdown on SKOV3 cell migration. Migration was assessed by determining the ability of cells to migrate in a culture plate using a wound-healing assay after 24 h of incubation. Abbreviations: sh-S3 and sh-S6 represent shRNA-mediated LY75 knockdown clones in SKOV3 cells; Ctrl represent the corresponding control SKOV3 cells.

A.

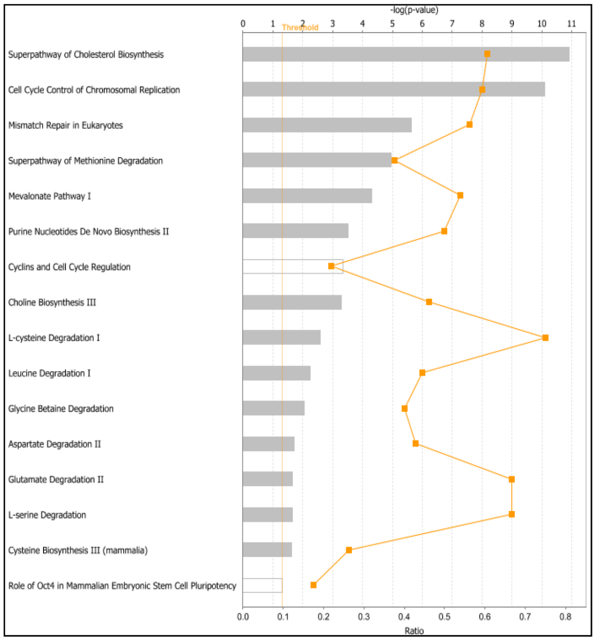

B.

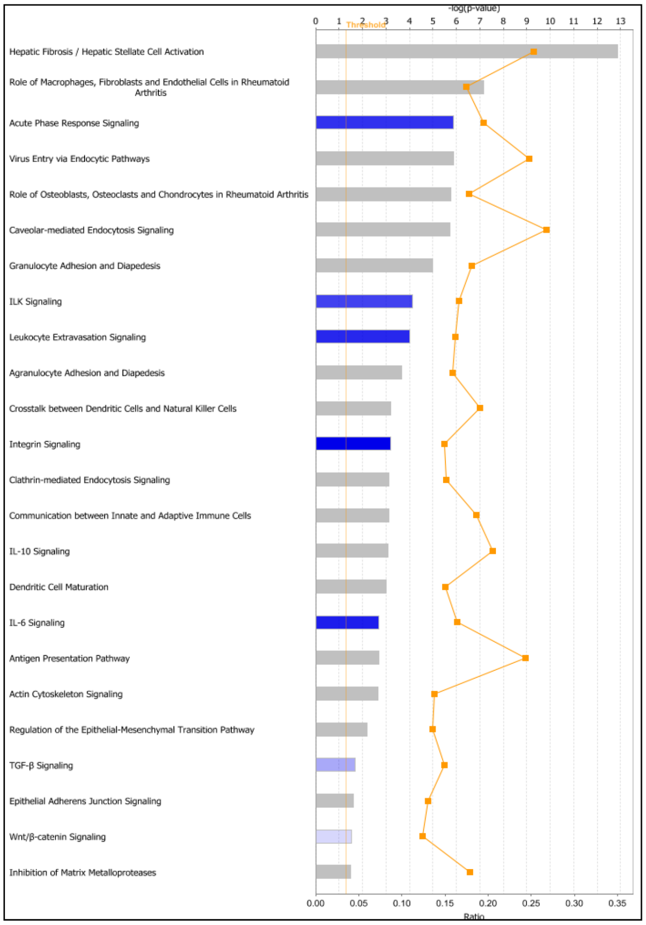

Supplementary Figure S6: Selected altered canonical pathways that were significantly dysregulated upon LY75 knockdown in SKOV3 cells. A. Upregulated canonical pathways; **B.** Downregulated canonical pathways. Top functions that meet a Fisher testing correction p-value of 0.05 are displayed.

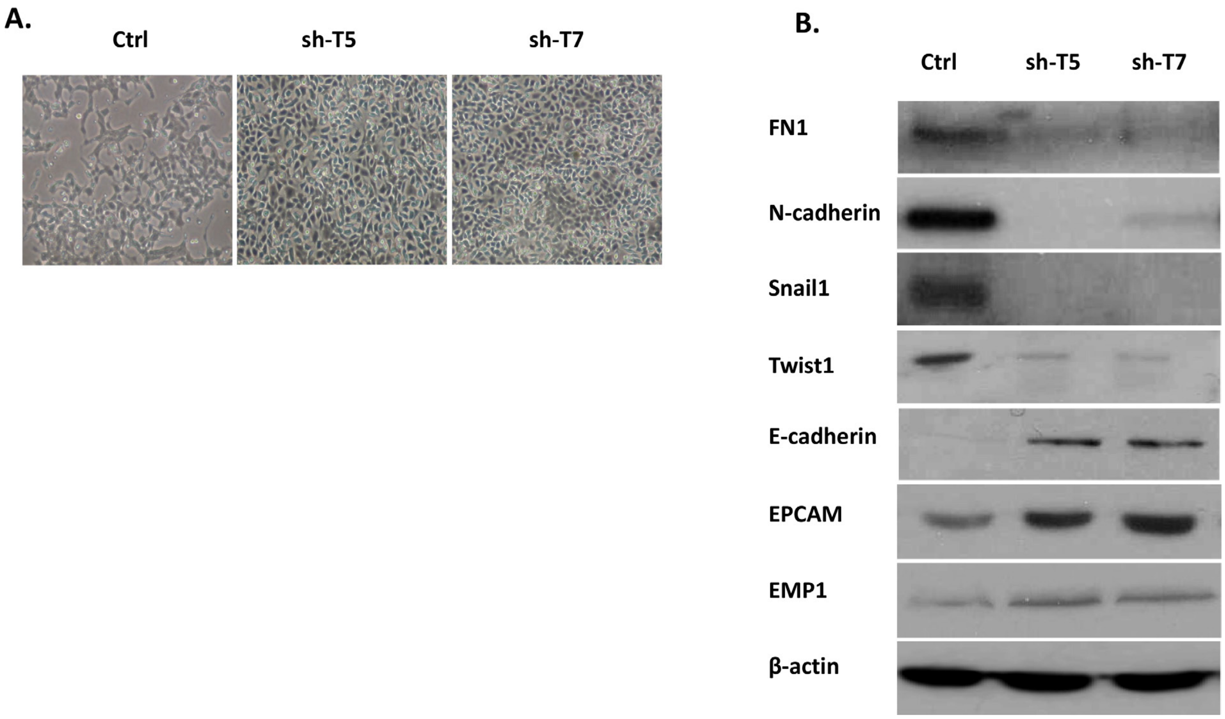

Supplementary Figure S7: shRNA-mediated LY75 suppression directs MET in TOV112 cells. A. Representative phase contrast images of TOV112 control and LY75 knockdown clones (sh-T5 and sh-T7). **B.** Western blot analysis of the expression of different EMT (epithelial and mesenchymal) markers in the control and the LY75 knockdown TOV112 clones.

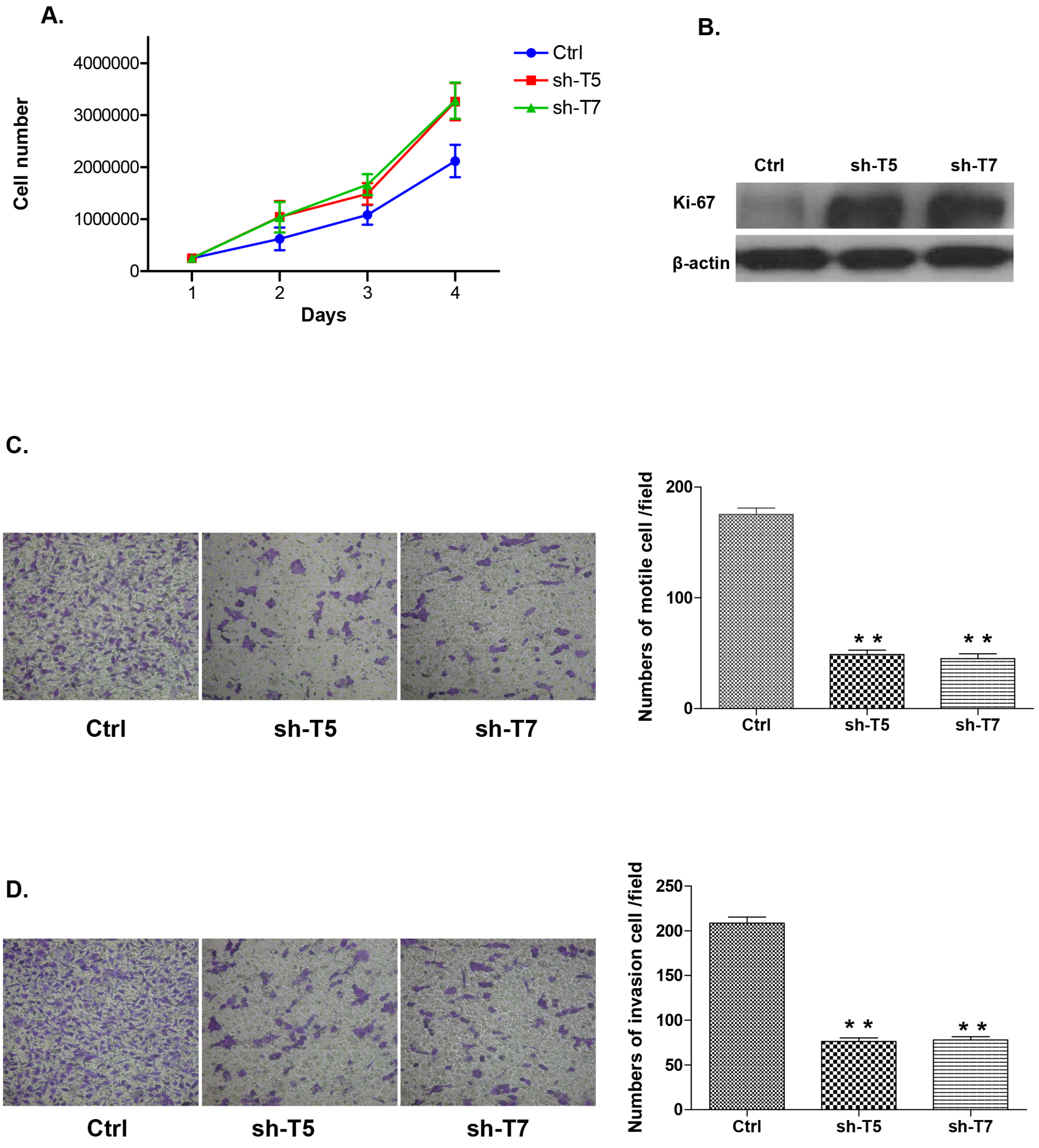

Supplementary Figure S8: Analyses of alterations in functional phenotypes upon shRNA-mediated LY75 knockdown in TOV112 cells. A. effect of LY75 knockdown on cell proliferation. **B.** Western blot analysis of the expression of the proliferation marker Ki-67 following LY75 knockdown. **C, D.** Representative images from one of the three independent experiments showing migration (C, left) and invasion (D, left) in the control clone and shRNA-LY75 clones sh-T5 and sh-T7 (at magnification ×400). The bar graphs in panels C (*right*) and D (*right*) are quantitative determinations of data obtained by selecting 10 random fields per filter (at magnification× 40) under phase contrast microscopy. Differences between shRNA-LY75-transfected and vehicle-transfected TOV112 cells were determined by a Student's t-test. Error bars denote ± SEM and *indicates statistical significance ($P < 0.05$).

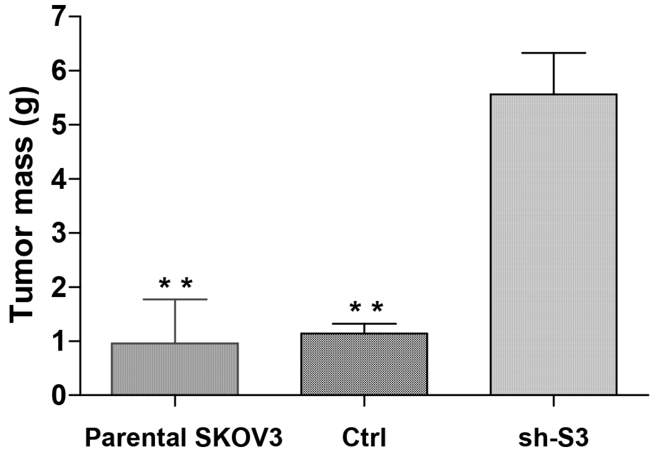

Supplementary Figure S9: Comparison of mean tumor mass from mice injected with the parental SKOV3 cells, mock-transfected (Ctrl) cells, and LY75 knockdown. Mice in the SKOV3 control & parental groups died due to respiratory distress, as 4 of the 16 mice in these experimental groups accumulated 2 ml or more ascites fluid. There was some sporadic tumor growth with tumor mass less than 1g. Mice in the SKOV3 LY75 knockdown group all died due to respiratory distress resulting from tumor bulk. Tumors were large and throughout the abdominal cavity; they tended to be well encased rather than invasive (with exception of 1 mouse with a liver tumor). Most of the tumors were arising within the mesentery, pancreas, omentum and lower abdominal fat pad. There was some diaphragm involvement but it wasn't huge and none of the mice developed ascites. Tumor mass is measured by removing all obvious tumor and weighing it. Differences between tumor mass in mice injected with parental SKOV3, Ctrl and sh-S3 cells were determined by a Student's t-test; error bars denote mean ± SEM; *indicates statistical significance ($p < 0.05$).

A. **B.**

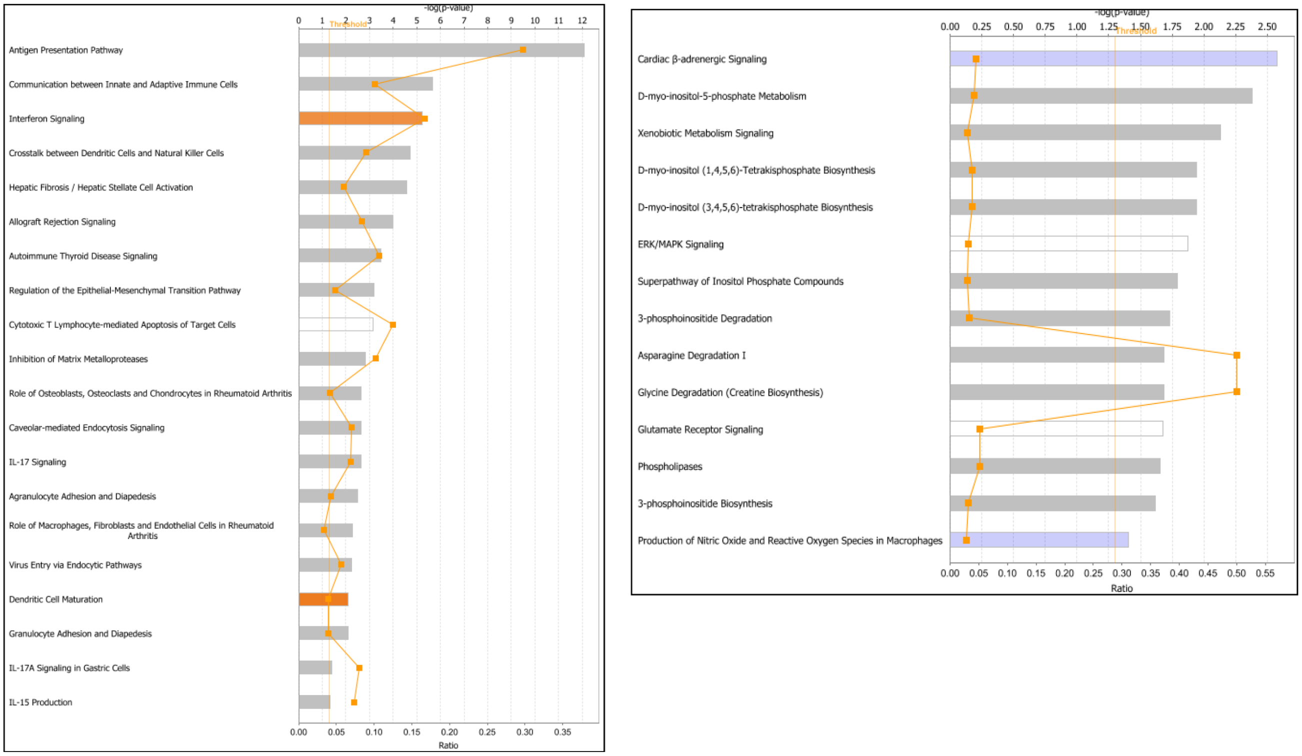

Supplementary Figure S10: Selected altered canonical pathways that were significantly dysregulated upon LY75 knockdown in A2780s cells. **A.** Upregulated canonical pathways; **B.** Downregulated canonical pathways. Top functions that meet a Fisher testing correction p-value of 0.05 are displayed.

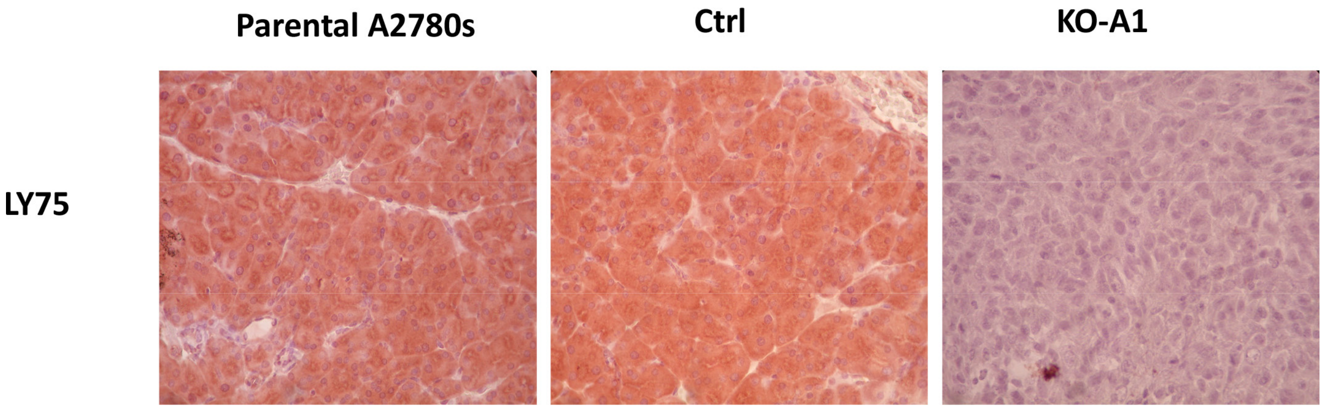

Supplementary Figure S11: Representative IHC images of LY75 expression in tumor tissues extracted from mice injected with the parental, Ctrl, and KO-A1 A2780s cells. Mice in all groups died due to respiratory distress resulting from tumor bulk. Tumors were large and throughout the abdominal cavity; Most of the tumors were arising within the mesentery, pancreas, omentum, liver, stomach and lower abdominal fat pad and reproductive organs (uterus and ovary).

Supplemental Table S1: Detailed Patients' clinicopathological characteristics

Variable	Range	n/total	%
Age (years)			
	≥65	64/130	49
	<65	66/130	51
Median	66		
Tissue/tumor type			
	Normal	13/130	10
	LMP	13/130	10
	High-grade	52/130	40
	OM	52/130	40
Grade			
	3	104/104	100
Stage			
	III	72/104	69
	IV	32/104	31
Chemotherapy[1]			
	platinum+taxol	97/104	93
	Other	13/104	7
CA125			
	≥800	47/104	45
	<800	53/104	55
PFS (months)[2]			
	0-6	41/103	40
	7-24	40/103	39
	> 25	22/103	21

[1]All patients were subjected to adjuvant therapy.
[2]Extended follow-up, including PFS values, were available for 103 patients.
OM - omental metastasis

Supplemental Table S2: Genes, members of the EMT and the TGF-β canonical pathways, downregulated upon LY75 knockdown in SKOV3 cells

See Supplementary File 1

Supplemental Table S3: Genes, differentially expressed in SKOV3 cells (≥ 2.0 fold, p≤0.05) following LY75 knockdown

See Supplementary File 2

Supplemental Table S4: Genes, differentially expressed in A2780s cells (≥ 2.0 fold, p≤0.05) following LY75 knockout

See Supplementary File 3
